# The lectin from *Musa paradisiaca* binds with the capsid protein of tobacco mosaic virus and prevents viral infection

**DOI:** 10.1080/13102818.2014.925317

**Published:** 2014-07-10

**Authors:** Xiao-Yu Liu, Huan Li, Wei Zhang

**Affiliations:** ^a^ Department of Biochemistry & Molecular Biology, College of Life Science, Nanjing Agricultural University, Nanjing, Jiangsu, P. R. China

**Keywords:** BanLec-1, capsid protein, *Musa paradisiaca*, *Nicotiana tabacum*, tobacco mosaic virus, viral infection

## Abstract

It has been demonstrated that the lectin from *Musa paradisiaca* (BanLec-1) could inhibit the cellular entry of human immunodeficiency virus (HIV). In order to evaluate its effects on tobacco mosaic virus (TMV), the *banlec-1* gene was cloned and transformed into *Escherichia coli* and tobacco, respectively. Recombinant BanLec-1 showed metal ions dependence, and higher thermal and pH stability. Overexpression of *banlec-1* in tobacco resulted in decreased leaf size, and higher resistance to TMV infection, which includes reduced TMV cellular entry, more stable chlorophyll contents, and enhanced antioxidant enzymes. BanLec-1 was found to bind directly to the TMV capsid protein *in vitro*, and to inhibit TMV infection in a dose-dependent manner. In contrast to limited prevention *in vivo*, purified rBanLec-1 exhibited more significant effects on TMV infection *in vitro*. Taken together, our study indicated that BanLec-1 could prevent TMV infection in tobacco, probably through the interaction between BanLec-1 and TMV capsid protein.

## Introduction

A wide array of abiotic and biotic stresses seriously affects plant growth and crop yield. Among the various biotic stresses, viral diseases represent one of the most significant reductions to crop quality and productivity.[1] When exposed to virus infection, plants show significant changes in photosynthetic efficiency, chlorophyll content, levels of reactive oxygen species, the activities of antioxidant enzymes and gene expression or RNA silencing.[2] The selection and cultivation of resistant varieties are the most cost-effective method to control plant virus diseases. Contrary to the classical breeding and marker-assisted selection approaches, direct transformation of specific genes into plants has been demonstrated to be one of the most effective ways for improving virus stress tolerance.[3,4]

Lectins are a huge protein family that recognize and bind to specific carbohydrates, and function as central mediators of information transfer in biological systems by interacting with glycoproteins, glycolipids and oligosaccharides.[5] Since an immune system is lacking in plants, it is generally suggested that most plant lectins are directed against foreign attacking organisms either in recognition or in defence-related processes, and may play a crucial role in the defence response against different pathogens (viruses, bacteria and fungi), phytophagous invertebrates and herbivorous animals.[6,7] On the basis of their carbohydrate-binding domain structure, plant lectins can be classified into 12 different groups.[8] One of these groups corresponds to the jacalin-related lectins (JRLs) characterized by the barrel-shaped-prism-I structure of the jack fruit seed lectin jacalin.[9] The JRL group is divided into two subfamilies, the man-JRL subfamily, which specifically binds mannose residues, and the gal-JRL subfamily, comprising those lectins with galactose-binding specificity.[10]

BanLec-1, a glucose/mannose-specific lectin, was isolated from banana and partially characterized.[11,12] It is a dimeric protein composed of two identical 15 kDa subunits. BanLec-1 has sequence similarity to JRLs, and is not only expressed specifically in fruit, but is also expressed developmentally during fruit ripening.[13] The crystal structure of BanLec-1 has a beta-prism-I fold, with two sugar-binding sites per subunit.[14] Based on this feature, it exhibits some uncommon binding properties as it recognizes 1,3-sugar units at the reducing termini, in addition to the internal α-1,3-linked glucosyl residues.[15]

BanLec-1 has been recognized as a potential immunomodulatory molecule.[16] Swanson et al. [17] further demonstrated that BanLec-1 could inhibit HIV-1 by binding directly to gp120, HIV-1 envelope protein, and was able to block HIV-1 cellular entry. In this study, we further characterized recombinant BanLec-1, and detected its antiviral activity against tobacco mosaic virus (TMV) in tobacco plants.

## Materials and methods

### Plasmid construction and tobacco leaf disc transformation

Banana (*Musa paradisiaca*) fruits were purchased from the local market. Total RNA was isolated by using Trizol reagent (Invitrogen, USA). The cDNA sequence was amplified by Reverse transcription polymerase chain reaction (RT-PCR). Primers for *banlec-1* cDNA (GI:37223481) were 5′ATGAACGGAGCGATCAAGGT and 5′TTATGGCTCCAAGTAGACCC. The amplified product was gel purified and cloned into binary vector pCAMBIA1304.1, which was then transformed into *Agrobacterium tumefaciens* strain GV3101. Leaf discs of *Nicotiana tabacum* NC89 were transformed. Transformed plants were selected on Murashige and Skoog medium (MS) [18] containing 100 μg/mL kanamycin and 50 μg/mL hygromycin B. After rooting, plants were transferred to soil and grown in the greenhouse (with 16/8 h light/dark) at 26 °C with 40%–50% relative humidity. After selection and growing in the field under natural conditions, T_2_ generation transgenic plants served as the material for this study.

### Gene expression verification in transgenic plants

Semi-quantitative RT-PCR was conducted by using primers as follows: *actin* F, 5′ TGGTTAAGGCTGGATTTGCT and *actin* R, 5′ TGCATCCTTTGACCCATAC; *banlec* F, 5′ ATGAACGGAGCGATCAAGGT and *banlec* R, 5′ TTATGGCTCCAAGTAGACCC. PCR products were analysed in a 1% agarose gel.

### Prokaryotic expression and purification

Total RNA was isolated by using Trizol reagent (Invitrogen, USA). PCR products were cloned into the expression vector pETGST and transformed into *E. coli* C43. For expression of recombinant BanLec-1 (rBanLec-1) with His_6_-tag at C-terminus, cells were grown in Luria–Bertani medium (LB) [19] containing 50 μg/mL kanamycin until an OD of 0.6–0.8 was achieved. rBanLec-1 expression was induced by adding isopropyl-β-D-thiogalactopyranoside (IPTG). The cell pellet obtained by centrifugation at 8000 ×*g* for 10 min was resuspended in 50 mmol/L NaH_2_PO_4_, 300 mmol/L NaCl, pH 8.0. After ultrasonic cell disruption, the suspension was centrifuged at 8000 ×*g* for 15 min. The supernatant was bound to high-affinity Ni-charged resin beads (GenScript, USA). The beads were washed in 50 mmol/L NaH_2_PO_4_, 300 mmol/L NaCl, 10 mmol/L imidazole, pH 8.0, and then rBanLec with His_6_-tag were eluted with 50 mmol/L NaH_2_PO_4_, 300 mmol/L NaCl, 250 mmol/L imidazole, pH 8.0, according to the manufacturer's instructions.

### Hem agglutination assay

Hem agglutination activity of the purified rBanLec was assayed in a 96-well microtiter U-plate. The protein samples and rabbit erythrocytes were diluted with phosphate-buffered saline (PBS), pH 7.4. At each well, 30 μL of sample from each dilution was mixed with 30 μL of 2% suspension of rabbit erythrocytes. The microtiter U-plate was kept at 37 °C for 1 h, and then hemagglutination was observed.

### Effect of metal ions on BanLec-1 activity

Purified rBanLec-1 at various concentrations (7.2, 3.6, 1.8, 0.9, 0.45, 0.22, 0.11 and 0.055 μg/mL) were incubated, respectively, with 5 mmol/L of different metal ions (Ca^2+^, Mg^2+^, Mn^2+^, Zn^2+^) overnight at room temperature. After that, 50 μL of a 2% (v/v) suspension of rabbit erythrocytes was added, and hem agglutination was observed after 1 h as described above, using at least three replicates for each assay.

### Thermal and pH stability assays

Purified rBanLec-1 was incubated at different temperatures ranging from 20°C to 90 °C for 30 min, or in buffers with different pH ranging from 1.4 to 12 at room temperature for 1 h, then hemagglutination activities were assayed as described above, and at least three replicates were done for each assay.

### TMV infection

TMV was prepared freshly by grinding 0.5 g of young systemically TMV-infected tobacco leaves in 2 mL of ice-cold 10 mmol/L PBS, pH 7.4. The resulting suspension was centrifuged at 100 g for 5 min at 4 °C. For *in vitro* analysis, TMV suspension was incubated with different concentrations (12.9, 4.28 and 1.71 μg/mL) of rBanLec-1 on ice for 30 min, and then inoculated in tobacco plants (cv. Huangyan and Samsun) mechanically after dusting with carborundum. Leaf tissue was collected at 48–72 h post inoculation. For *in vivo* analysis, the leaves of transgenic and wild-type (NC89) lines at the stage of 6–7 leaves were mechanically inoculated after dusting with carborundum. Leaf tissue was collected at 0, 6, 12, 18 and 24 days post inoculation. All experiments were carried out in triplicate. Disease rate (%) was calculated as [number of infected plants / number of tested plants] × 100), and disease index (%) was calculated as {∑[Disease progression × numbers of plants of corresponding disease progression] / [The highest disease progression×number of tested plants]}×100).

### Chlorophyll content measurement

To measure the chlorophyll content, 0.5 g of tobacco leaf filaments were soaked in 5 mL of 80% (v/v) acetone until the colour of the filaments completely changed from green to white. The supernatant of the extract was re-set to 5 mL and taken for light absorption measurement at 663 nm and 645 nm, respectively.

### Ultrastructure observation of leaf cells

Rectangular segments (1.5 mm × 1 mm) were cut from the leaves of transgenic and wild-type (WT) lines between the third and the fourth principal vein. The segments were fixed for 2 h in 2% glutaraldehyde, then washed in PBS buffer three times, and fixed again in 1%–2% OsO_4_ for 2–3 h. After fixation and dehydration by ethanol, leaf samples were washed by acetone for three times, and embedded in Spurr's resin. Ultrathin sections were sequentially stained with uranyl acetate and lead citrate, followed by examination under a transmission electron microscope.

### Activities of antioxidant enzymes

Leaves of WT and transgenic lines were grinded in ice cold 50 mmol/L PBS (pH 7.8) containing 0.5% Triton X-100, 10 mmol/L β-mercaptoethanol, 0.1 mg/mL serine proteinase inhibitor (PMSF). Crude tissue homogenates were centrifuged at 12,000 ×*g* for 10 min at 4 °C. The supernatants were used for determination of superoxide dismutase (SOD) and peroxidase (POD) activities.[20]

### Gel overlay assay

Purified rBanLec-1 and whole cell extracts after IPTG induction were separated by sodium dodecyl sulphate polyacrylamide gel electrophoresis (SDS-PAGE) and electro-transferred onto polyvinylidene difluoride (PVDF) membrane. The membrane was then washed for 15 min in buffer A (30 mmol/L Tris–HCl, pH 7.4, 0.05% Tween 20). The washed membrane was denatured for 2 h at room temperature in denaturation buffer (6 mol/L guanidine hydrochloride, 2 mmol/L ethylenediaminetetraacetic acid (EDTA), 50 mmol/L dithiothreitol (DTT), 50 mmol/L Tris–HCl, pH 8.3). After a brief wash in tris-buffered saline (TBS: 140 mmol/L NaCl, 30 mmol/L Tris–HCl, pH 7.4) and renaturation buffer (140 mmol/L NaCl, 10 mmol/L Tris–HCl, pH 7.4, 2 mmol/L EDTA, 1% bovine serum albumin (BSA), 0.1% Tween-20, 2 mmol/L DTT), the blotted proteins were renatured in renaturation buffer overnight at 4 °C, and then the PVDF membrane was blocked with 1% BSA in renaturation buffer for 90 min at room temperature. For TMV capsid protein (CP) binding, the membrane was incubated for 90 min at room temperature with 10 μg/mL of TMV CP in the renaturation buffer. After incubation, the membrane was washed four times with TBST (TBS plus 0.1% Tween-20) and incubated for 1h at room temperature with rabbit anti-TMV CP antiserum (1:1000 dilution) for 1 h. After four washes with TBST, the membrane was detected with horseradish POD and immunoglobulin G (HRP-IgG).

## Results and discussion

### Expression, purification and characterization of recombinant BanLec-1

**Figure 1.**
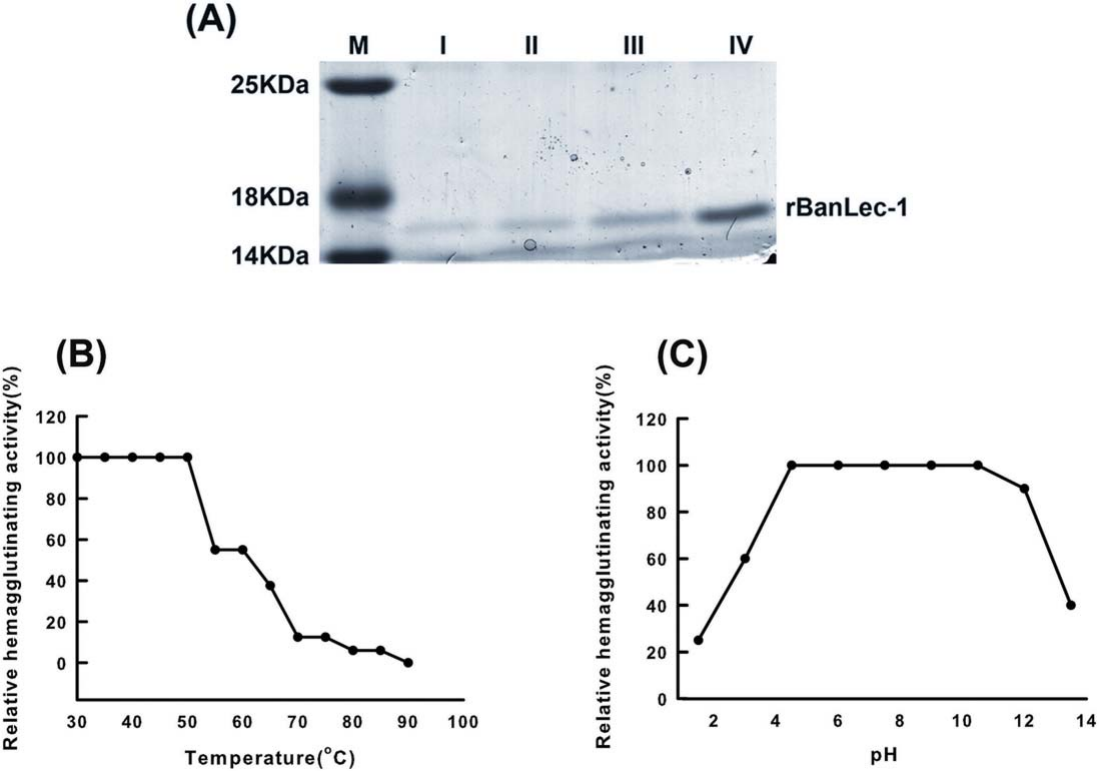
Expression, purification and characterization of BanLec-1. Expression and purification (A) of rBanLec-1 in *E.coli* CP43: M, protein Marker; I, sample induced by IPTG for 0.5 h; II, sample induced by IPTG for 1 h; III, sample induced by IPTG for 2 h; IV, sample induced by IPTG for 3 h. Thermal stability of purified rBanLec (B) at different temperatures ranging from 20 °C to 90 °C for 30 min; hem agglutination activities were assayed. pH stability of purified rBanLec (C) at pH ranging from 1.4 to 12 at room temperature for 1 h; hem agglutination activities were assayed.

The *banlec-1* gene (GI:37223481) was cloned and the resulting protein with His_6_-tag was expressed in *E. coli* C43 under IPTG induction. After purification through high-affinity Ni-charged resin, the purified product was analysed by 12% SDS-PAGE and the appearance of an approximately 15 kDa band in the gel indicated the purity of rBanLec-1 after 3 h of IPTG induction (Figure 1(A)). The effects of temperature and pH on the activity of rBanLec are shown in Figure 1(B) and 1(C), respectively. The agglutination activity was stable up to 50 °C, while 50% of the activity was lost at 60 °C, and there was less than 12.5% of activity left at 70 °C. The results are consistent with the differential scanning calorimetry (DSC) analysis that BanLec-1 had a transition maximum temperature (*Tm*) of 60.8 °C.[21] The agglutination activity of rBanLec was stable in a broad pH range from 4 to 12, and was gradually lost when pH values were below 4 or above 12, which indicated that rBanLec was stable under alkali conditions. These results supported the observations that BanLec-1 is a stable dimer in solution,[12] and the stability of the dimer may result from strong hydrogen bonds and water bridges at the interface between the two subunits of BanLec-1.[21] The effect of divalent cations on rBanLec-1 activity was evaluated with four different metal ions. Ca^2+^, Mg^2+^ and Mn^2+^ were found to be able to support agglutination, whereas Zn^2+^ was not (Table 1). Many lectins have been reported to be metalloproteins, and this is the first report that the activity of BanLec-1 is dependent on metal ions.

**Table 1.** Metal ions dependence of the recombinant BanLec-1.

	BanLec concentration (μg/mL)							
Reagents	7.2	3.6	1.8	0.9	0.45	0.22	0.11	0.055
Ca^2+^	+	+	+	−	−	−	−	−
Mn^2+^	+	+	+	+	+	−	−	−
Mg^2+^	+	+	+	+	+	−	−	−
Zn^2+^	−	−	−	−	−	−	−	−

Note: Concentration of metal ions was 5 mmol/L. +, hem agglutination; −, no hem agglutination.

### Overexpression of BanLec-1 in tobacco confers higher resistance to TMV infection

**Figure 2.**
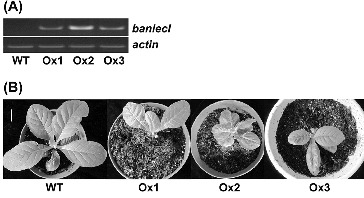
Construction of transgenic tobacco plants. Analysis of *banlec-1* expression levels (A) in different transgenic tobacco lines by semi-quantitative RT-PCR: OX1–OX3, independent transgenic tobacco lines. Phenotypes (B) of wild-type (WT) and transgenic tobacco seedlings (OX1, OX2, OX3). White bar = 1.5 cm.

In order to verify the BanLec-1 effects on TMV infection, we constructed *banlec-1* overexpressing transgenic tobacco plants. Semi-quantitative RT-PCR results showed that although *banlec-1* was expressed in all tested transgenic lines, the expression level of individual lines was different (Figure 2(A)), and the phenotype analysis indicated that transgenic plants had reduced leaf size compared to WT (Figure 2(B)). For chlorophyll contents, however, there were no significant differences between transgenic and WT plants under normal growth conditions (data not shown).

**Figure 3.**
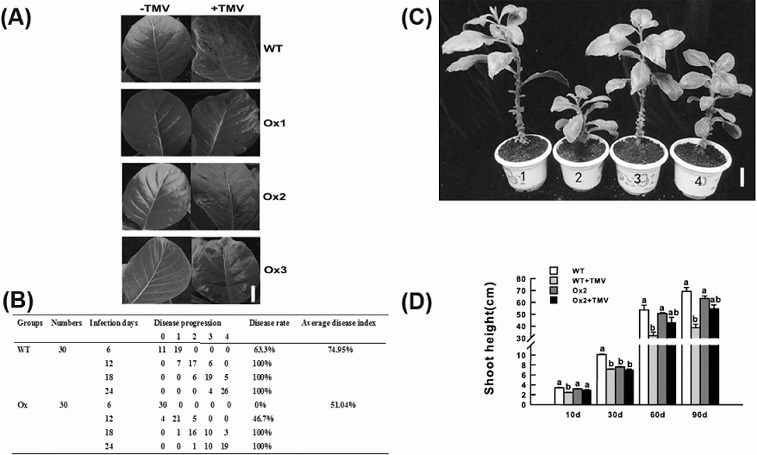
Phenotype analysis in wild-type (WT) and transgenic plants after TMV infection. Hypersensitive reaction syndrome (A) in the leaves of transgenic (OX1, OX2, OX3) and WT tobacco plants with or without TMV infection: white bar = 1 cm. Statistical analysis and comparing of relevant indicators (B) between transgenic and WT tobacco plants after TMV infection. Shoot height changes (C) in transgenic and WT tobacco plants after TMV infection: 1, WT; 2, WT with TMV infection; 3, OX2; 4, OX2 with TMV infection; white bar = 5 cm. Statistical analysis of shoot height (D) in transgenic and WT tobacco plants. Same letters indicate no significant difference; different letters indicate significant differences.

As expected, the overexpression of BanLec-1 alleviated TMV infection syndrome in tobacco leaves (Figure 3(A)). The results indicated that during the first 12 days after infection, the disease rate in the transgenic plants was significantly lower than that in the WT ones, and the average disease index in OX2 was 51.04%, whereas it was 74.95% in WT lnes (Figure 3(B)). Although both the transgenic and WT plants showed dwarf phenotypes after infection, WT plants were much shorter than OX2 (Figure 3(C) and 3(D)).

**Figure 4.**
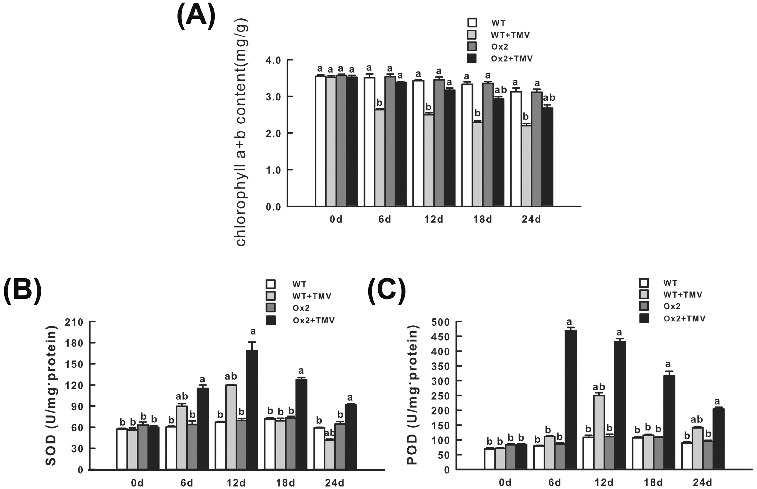
Physiological analysis of wild-type (WT) and transgenic tobacco plants (OX2) after TMV infection: total chlorophyll content (A), SOD activity (B) and POD activity (C) in leaves.

It has long been known that TMV infection would result in the decrease of chlorophyll content, probably through the suppression of key genes involved in chlorophyll biosynthesis by TMV-induced gene silencing.[22] In this study, after infection with TMV, the chlorophyll contents were found to be continuously decreased in both OX2 and WT lines. Nevertheless, the extent to which the chlorophyll content changed in OX2 was much lower than that in WT plants. For example, 12 d after inoculation with TMV, the total chlorophyll contents of WT plants decreased to 74.4% of their normal level, whereas those of the transgenic lines were still 93.2% of their normal levels (Figure 4(A)), indicating that overexpression of BanLec-1 in tobacco prevented the decrease of chlorophyll which was induced by TMV infection. Without TMV treatment, the activities of POD and SOD showed no obvious difference between WT and OX2. After TMV infection, the activities of SOD and POD were significantly increased in the OX2 line, while they were just mildly increased in the WT plants (Figure 4(B) and 4(C)).

**Figure 5.**
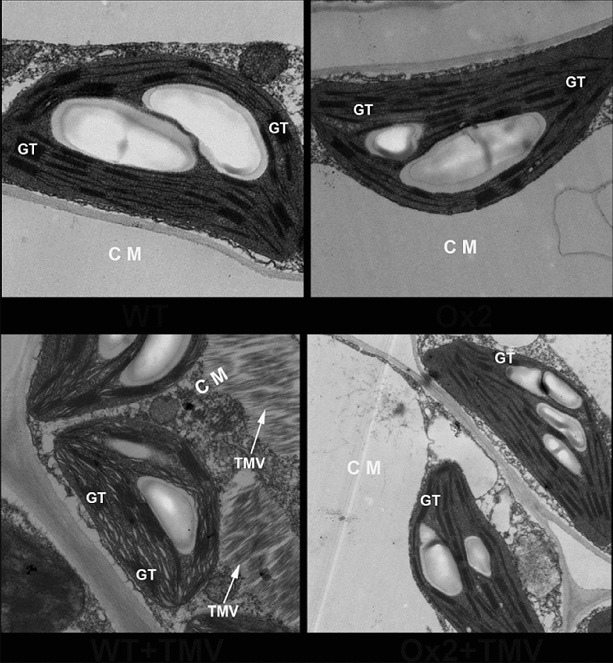
Ultrastructure of OX2 and WT tobacco leaves with or without TMV infection for 10 days. GT: grana thylakoids; CM: cytoplasmic matrix. White arrow indicates TMV particles.

We further investigated whether BanLec-1 overexpression *in vivo* could also prevent TMV cellular entry. As shown in Figure 5, after infection for 10 days, a large number of TMV particles were observed in the cytoplasmic matrix in WT cells (white arrows), while there were almost no detectable TMV particles in the cytoplasmic matrix in OX2. Compared with OX2, the damage of grana thylakoids (GT) was also more serious in WT. Taken together, these results demonstrated that overexpression of BanLec-1 confers higher resistance to TMV infection in tobacco.

### BanLec-1 prevents TMV infection by binding with TMV capsid protein

**Figure 6.**
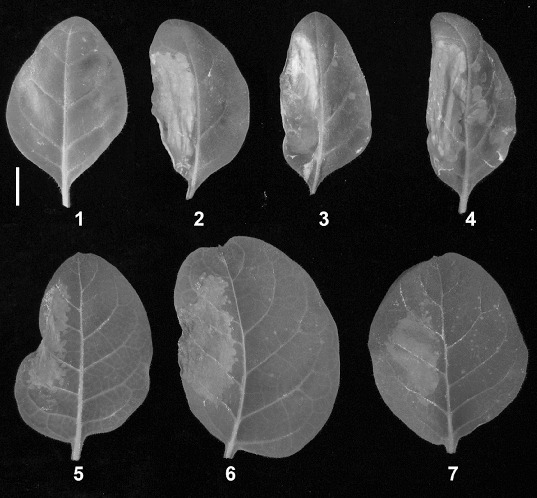
*In vitro* analysis of the inhibitory effect of BanLec-1 on TMV infection. Upper panel: *Nicotiana tabacum* cv. Huangyan; lower panel: *Nicotiana tabacum* cv. Samsun. Left part of the leaf: 1, PBS buffer; 2–7, TMV suspension; right part of the leaf: 1, 12.9 μg/mL of rBanLec-1 alone; 2–4, TMV suspension with 12.9 μg/mL, 4.28 μg/mL, and 1.71 μg/mL of rBanLec-1, respectively; 5–7, TMV suspension with 12.9 μg/mL, 4.28 μg/mL, and 1.71 μg/mL of rBanLec-1, respectively. White bar = 2 cm.

**Figure 7.**
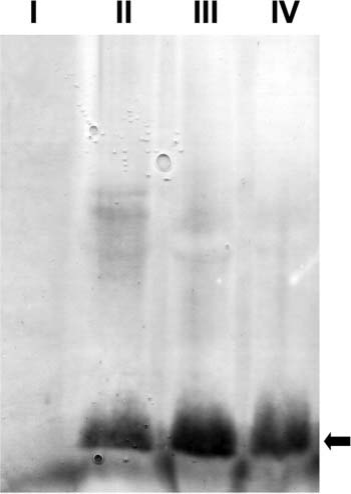
Gel overlay assay. I, 0.2% BSA; II, *E.coli* whole cell lysate after IPTG induction; III, 12.9 μg/mL of rBanLec-1; IV, 4.28 μg/mL of rBanLec-1. Black arrow indicates the 15 KDa bands.

It has been reported that BanLec-1 could bind with human immunodeficiency virus (HIV) coat protein gp120 and prevent HIV infection.[17] At the same time several studies showed that the lectins from different resources could interact with the coat or envelope proteins of different viruses.[13,23,24] In order to reveal the mechanisms by which BanLec-1 conveys resistance to TMV infection, purified rBanLec-1 protein at different concentrations were incubated with TMV at 4 °C for 30 min, and then inoculated to tobacco leaves. As shown in Figure 6, inoculation of BanLec-1 alone had no effects on leaf growth in different tobacco varieties (*Nicotiana tabacum* var. Huangyan and Samsun NN). In contrast, pre-incubation *in vitro* effectively inhibited TMV infection in a dose-dependent manner, and 12.9 μg/mL of rBanLec-1 inhibited TMV infection completely. Gel overlay assay indicated that IPTG induced whole cell extracts and purified rBanLec-1 at different concentrations could bind specifically with TMV CP. The horse radish peroxidase (HRP) signal bands exhibited at about 15 KDa further demonstrated that BanLec-1 could interact with TMV CP directly (Figure 7).

These results demonstrated that BanLec-1 may prevent TMV infection, and maintain relatively normal physiological parameters such as chlorophyll content, photosynthetic rates and enhanced antioxidant activities. However, such *in vivo* prevention was limited, i.e. once TMV replicates itself in cells and its content exceeds the effective concentration of BanLec-1, infection still occurs. That is why the disease rate remained at zero in transgenic plants in the first 6 d after infection, and reached 100% after 18 d (Figure 3(B)).

In contrast to limited prevention *in vivo*, purified rBanLec-1 exhibited more significant effects on TMV infection *in vitro*. At a concentration of about 13 μg/mL, BanLec-1 almost completely inhibited TMV infection (Figure 6), and gel overlay assay further demonstrated that BanLec-1 could bind to TMV CP (Figure 7). Since TMV CP is an unglycosylated protein, these results support the idea that BanLec-1 binds to TMV particles through protein–protein interaction between BanLec-1 and TMV CP. These findings, combined with higher structural stability (Figure 1(B) and  1(C)) and lower molecular weight (Figure 1(A)) of BanLec-1, make it a potential candidate for the research and development of protective agents against plant viral diseases. In order to achieve this aim, further studies are needed to evaluate whether BanLec-1 has similar effects on other plant viruses.

## Conclusions

BanLec-1, the lectin from *Musa paradisiaca*, was shown to be a stable glycoprotein whose activity is dependent on metal ions. Overexpression of BanLec-1 in tobacco resulted in resistance to TMV infection both *in vitro* and *in vivo*. In contrast to limited prevention *in vivo*, purified rBanLec-1 exhibited more significant effects on TMV infection *in vitro*. This is the first report that BanLec-1 could prevent TMV infection in tobacco, probably through the interaction between BanLec-1 and TMV CP. 

## Funding

This work was supported by the Fundamental Research Funds for the Central Universities [grant number KYT201001] and a project funded by the Priority Academic Program Development of Jiangsu Higher Education Institutions (PAPD), Jiangsu, China.
